# Level of Partograph completion and healthcare workers’ perspectives on its use in Mulago National Referral and teaching hospital, Kampala, Uganda

**DOI:** 10.1186/s12913-019-3934-3

**Published:** 2019-02-07

**Authors:** John Mukisa, Isha Grant, Jonathan Magala, Andrew S. Ssemata, Patrick Z. Lumala, Josaphat Byamugisha

**Affiliations:** 1 Uganda CWRU Research Collaboration, Kampala, Uganda; 2 Department of Child Health, Ministry of Health, Kampala, Uganda; 3 Department of Obstetrics and Gynecology, Life link hospital, Kampala, Uganda; 4 Department of Psychiatry, Makerere University College of Health Sciences, Kampala, Uganda; 5 Research and Planning Division, Uganda Revenue Authority, Kampala, Uganda; 6 Department of Obstetrics and Gynecology, Makerere University College of Health Sciences, Kampala, Uganda

**Keywords:** Partograph, Healthcare worker perspectives

## Abstract

**Background:**

The appropriate use of the Partograph allows early identification of labour related complications and prevents deaths. We, therefore, sought to determine the level of Partograph completion and healthcare worker perspectives towards its utilization.

**Methods:**

This study had two components; a hospital-based cross-sectional descriptive chart review at the Mulago National Referral Hospital, Kampala, Uganda and a qualitative study involving four Focus Group Discussions (FGDs) with ward nurses, midwives and postgraduate residents. Data from the FGDs were analyzed using thematic -content analysis in Open Code software. The quantitative data were summarized using descriptive statistical analysis, means and proportions.

**Results:**

Among the 355 Partographs reviewed, 79.1% had incomplete documentation of age, 52.7% gravidity, and 3.2% parity. In about 61%, the specific parameters for fetal monitoring, maternal monitoring and labour progress were incomplete. From the FGDs, the healthcare workers reported being unable to complete the Partographs due to the overwhelming numbers of expectant mothers and other staff responsibilities. Congestion in the maternity ward reduced the Partograph completion rates. The availability of other monitoring tools, limitation in skills, inadequate equipment and supplies, and the state of the mother at the presentation to the hospital all made Partograph use and completion challenging.

**Conclusions:**

The majority of Partographs started by health workers were incomplete. The time required to document, health system challenges, status of mother at presentation, and the high workload undermined completion of the Partograph at this high volume facility.

## Background

Every day, approximately 1000 women die from preventable causes related to pregnancy and childbirth, with 99% of these maternal deaths occurring in low and middle-income countries [1]. The highest burden is in the regions of Sub-Saharan Africa and parts of Asia [2, 3]. In Uganda, estimated maternal mortality ratio in 2016 was 336 per 100,000 live births (a decrease from 438/100,000 in 2011) with a Ugandan women’s lifetime risk of maternal death standing now at 1 in 53 [4–6].

According to World Health Organization (WHO), one of the key important requirement for averting these deaths is the provision of care by a skilled birth attendant before, during and after childbirth [7]. Skilled birth attendant care needs to be available across all levels of the health system in order to reduce the delays for a referral to a higher care level if problems are expected to arise or do arise during labour. The Partograph is also used in conjunction with this intervention. The Partograph is a graphical record of the progress of labour and relevant details of the mother and the fetus [8]. It has action and alert lines to stimulate commencement of additional interventions by a skilled birth attendant monitoring the progress of labour [9].

The Partograph is recommended by the WHO as a means to monitor and record maternal and fetal well-being as it can identify maternal or fetal distress, and abnormalities in the progress of labour that require further action, including referral [10–13]. The appropriate utilization of the Partograph is one of the core skills of a trained birth attendant [9]. This can reduce complications from prolonged labour for the mother: female genital fistula, postpartum hemorrhage, sepsis, uterine rupture and its sequelae; and for the infant: death, anoxia, and infections.

Despite documentation of the benefits of Partograph completion, challenges to the adoption of this labour tool have been recognized in low and middle-income countries [14]. In Uganda, the Ministry of Health introduced the use of the Partograph in managing labour in the early 1990s although its utilization has been low with reported rates of 69.9 and 15.7% in Rujumbura health sub-district and Bwera hospital respectively [15, 16]. A number of factors contributed to the low Partograph utilization and completion according to recent studies. These include the large numbers of deliveries made without skilled and trained personnel [17]; lack of knowledge about proper Partograph utilization, unavailability of the Partographs and absence of on the job training [12, 15, 18]. However, most of these studies were restricted to rural health facilities, with few highly skilled healthcare personnel which might have biased Partograph use estimates and overlooked health worker perceptions. In this paper, we present the findings from a study that determined the rate of completion of Partographs in Mulago National Referral and teaching Hospital in Kampala, Uganda. We also explored the perceptions of health care workers involved in maternal deliveries concerning the usefulness of the Partograph and impediments to its utilization and completion in this setting.

## Methods

### Study design

We carried out a cross-sectional study between 1st March and 31st May 2016 that combined quantitative and qualitative methods of data collection. The exploratory qualitative component employed four Focus Group Discussions (FGDs) as the primary data collection method (5). The quantitative component used a retrospective selective cluster randomized chart review of Partograph use in deliveries during the study period.

### Study setting

The study was conducted at Mulago National Referral and teaching Hospital in Uganda. The hospital is situated 2.5 km by road to the north of Uganda’s capital, Kampala, and serves the catchment-area of 20 km radius which has low to middle income population. The hospital provides antenatal, intrapartum and postnatal services and has inpatient facilities for obstetric conditions. Annually approximately 33,000 deliveries are conducted making it one of the busiest maternity units in the world [19]. Every month approximately 2750 women deliver on one of the three labour wards (two public, one private), many after referral because of complications. Each day, the labour wards receive 60–80 admissions, deliver about 50–70 babies by vaginal route, 20–25 and 2–5 babies by emergency and elective caesarean deliveries respectively [17]. The wards are run by a staff of 46 midwives supported by intern doctors, obstetricians and postgraduate doctors training to be obstetricians. The ward coverage has an average of 5–6 personnel attached to each of the three shifts per day in every ward. The health worker to patient laboring ratio reaches 1:15 during peak delivery hours of each shift on the busiest ward. This hospital was chosen as the study site since it has the highest delivery rates in Uganda and has different cadres of health care workers who provide care for women admitted for delivery.

### Study population

For the quantitative component, we carried out a retrospective chart review of selected Partographs conducted between 1st March and 31st May 2016. Partographs for women who were admitted in labour at any gestational age cervical dilatation of any degree on admission if delivered in the months of data collection were eligible for review irrespective of the eventual mode of delivery. The sample size for the number of charts to review for Partograph completion was determined using the Kish & Leslie formula [*n* = (Zα/2)^2^ P (1-P)/ d^2^; whereas, Z: significance level, P: proportion (prevalence), and d: marginal error]. We assumed a 65.3% estimated proportion of the Partographs use and left incomplete obtained from a study done in Malawi [20]. We considered a 5% of the marginal error and a two-tailed at 95 confidence interval Z value of 1.96. The minimum estimated sample size determined was 352. Clinical charts for patients discharged from labour wards during the study period were selected by systematic random sampling. The sampling interval was obtained by dividing the required sample size by the number of files for patients discharged during the study period. Every day, the total number of deliveries in each of the wards were counted. The total number of patient charts with a Partograph ever started on each ward was determined. The position two was then selected as the starting point for the interval sampling. The selected charts were then reviewed by the study team to determine the level of completeness of the Partographs at each ward until a sample size was assessed.

For the qualitative component, we purposively identified health workers (nurses, midwives, obstetricians, postgraduate doctors training in obstetrics and gynaecology) both on day shift or night shift at the time of data collection from the three labour wards to participate in one of the four planned Focus Group Discussions (FGD). The selected healthcare personnel needed to have spent more than 6 months in the maternity section of the hospital to participate. We excluded health workers working in other departments and workers on holiday or on leave who work in the labour wards. Each FGD was composed of eight participants with planned homogeneity with respect to the ward to encourage participation and to allow an analysis based on commonality of experience in the profession for example nurses and midwives (two FGDs); obstetricians (one FGD), postgraduate doctors training in Obstetrics and Gynaecology (one FGD). At the end of the four FGDs and qualitative data review, the data saturation point had been reached and no additional FGDs were conducted.

### Data collection tools

#### For quantitative

Selected files were retrieved with the help of the ward record clerks. Data were collected on the number of admission per day to facilitate sampling of the charts. The information on the level of Partograph use and completion was abstracted onto the study questionnaire.

#### For qualitative

Written informed consent was obtained for each participant in the FGD. The FDGs were moderated by a trained research assistant, audio recorded and continued on until no new ideas were being imparted (data saturation). We used a FGD guide that had been developed by the multidisciplinary team of an obstetrician, public health specialist, an epidemiologist and social scientist. It was written in English and administered by trained research assistants (in qualitative research methods), under the supervision of the Principal Investigator to elicit data. We explored specific factors or reasons that prevented health workers from utilizing and filling in the Partograph through examining their experiences and identifying issues that confronted them collectively as well as individually. All FGDs were conducted in English, the commonly spoken language by the interviewees.

### Data analysis

The extracted data from the chart and Partograph reviews were entered into Epidata Version 3.1 with programmed logic checks. Data was then exported and analyzed using SPSS version 16.0. Data cleaning, clarification and quality checks were done before data analysis. Descriptive statistics and frequency tables were presented to describe the extent to which the Partograph was being utilized. The three key parameters of fetal monitoring, labour progress and maternal condition were assessed as follows: fetal heart rate monitoring (FHR), the status of membranes, liquor, and moulding. Maternal condition: temperature, blood pressure, and pulse rate, and labour progress: cervical dilatation, uterine contractions, and descent of fetal head. The Alert line if crossed and action line if reached were also assessed. Other pieces of information collected included: the home address, date of admission, date and time of labour onset, age, date and time of membranes ruptured and abnormal symptoms, details of the first examination on admission, first vaginal examination, pelvic assessment, and time of the second stage of labour. Personal details such as the name of the mother were not collected.

The extent of the Partograph completion was assessed by determining the proportion of the sections filled based on the following: *Complete*: if all the three components (fetal monitoring, labour progress and maternal monitoring) were filled out. *Adequately* filled in: if the three components had some information even if lacking in some parameters. Inadequately filled in: if only two components were filled out. *Grossly inadequate*: If only one component was filled out. *Blank*: if there no part or sections of the Partograph were filled.

The FGDs were transcribed verbatim and analyzed thematically by an experienced social scientist using standard principles [21]. The field team, together with the authors of the paper under the leadership of the principal investigator read through the transcripts and notes from the discussions several times, identifying meaning units. The meaning units were then condensed, coded and put into categories, from which themes emerged. We used Open code software to analyze the qualitative data as well [22].

## Results

### Quantitative findings

We conducted a retrospective review of 355 files from the three labor wards in Mulago National Referral Hospital. About 60% (212/355) of the charts were obtained from the busiest ward among the three delivery wards. This was the most utilized public ward while the rest of the charts were obtained from the other smaller public ward 26% (94/355) and the private delivery ward 14% (49/355). Of the selected charts for review, none was missing from the hospital records team likely because of the short duration between previous use and storage.

### Partogragh review

#### Background contents of the Partograph-

As shown in Table 1, among the 355Partographs reviewed, over 50% of them were incomplete in key findings. In 79.1, 52.7, and 53.2% of the Partographs, the mothers’ age gravidity, and parity, respectively had not been entered.

**Table 1:** The level of Completeness of Socio demographic and admission characteristics on the Partographs reviewed between March and May 2016

Characteristic on the Partograph	Complete, N (%)	Incomplete, N (%)	Missing, N (%)
Name of patients	166 (46.7)	187 (52.7)	2 (0.5)
Address	51 (41.4)	303 (85.3)	1 (0.3)
Age	73 (20.6)	281 (79.1)	1 (0.3)
Gravidity	167 (47.0)	187 (52.7)	1 (0.3)
Parity	165 (46.5)	189 (53.2)	1 (0.3)
Time of admission	158 (44.5)	196 (55.2)	1 (0.3)
Date and onset of labour	109 (30.7)	236 (65.5)	10 (2.8)
Date and time membranes ruptured	112 (31.5)	241 (67.9)	2 (0.6)
Has abnormal symptoms	15 (4.2)	338 (95.2)	2 (0.6)

### Maternal, fetal and labour documentation in the Partograph

#### The degree of overall completeness by ward

Overall only 8.8% of the Partographs for these 355 women were adequately completed but for the busiest public ward, this dropped to only 5.2% (Table 2).

**Table 2:** Table showing completeness of all sections of the Partographs on all the 3 wards

Completeness	Busiest public ward	Second public ward	Private ward	Total
	n,%	n, %	n, %	n, %
Complete	5 (2.3)	61 (64.9)	21 (42.9)	87 (24.6)
Adequately filled in	11 (5.2)	7 (7.4)	14 (28.6)	32 (8.8)
Inadequately filled in	10 (4.7)	5 (5.3)	6 (12.2)	21 (5.6)
Grossly inadequate	186 (87.7)	21 (22.3)	8 (16.3)	215 (60.6)
Total	212	94	49	355

### Completeness of documentation on the Partograph

As shown in Table 3, the three sections (fetal monitoring, labour progress, and maternal monitoring) on the Partograph had different rates of documentation with the maternal monitoring parameters having the lowest (25.7%) and fetal monitoring the highest (33.7%.). Given the low overall rate of completion, we did not consider the time of day. Within each category, major omissions were identified. For instance, for the labour progress section, only 5.6% recorded “Alert line” and in Maternal Monitoring, the temperature was only recorded 11.8% of the time.

**Table 3:** The level of completeness of Partographs

	Complete (%)	Incomplete (%)	Missing (%)
Fetal Monitoring			
Fetal heart rate	39.4	60.3	0.3
Liquor	32.4	67.3	0.3
Moulding	29.3	70.4	0.3
Average	33.7	66	0.3
Labor progress			
Cervical dilation	42	57.7	0.3
Descent	37.7	62	0.3
Contraction	36.9	62.8	0.3
Alert line	5.6	90.7	3.7
Average	30.6	68.3	1.2
Maternal monitoring			
BP	37.2	62.5	0.3
Pulse	28.2	71.5	0.3
Temperature	11.8	87.6	0.6
Average	25.7	73.9	0.4

### Qualitative findings

Thirty two out of the 35 participants approached agreed to participate in the FGDs. In total, 6 nurses, 10 midwives, 13 postgraduate students in obstetrics and gynaecology, 3 obstetricians and gynaecologists with at least 2 years of practice after graduate training in obstetrics and gynaecology degree studies, participated in the four FGDs. Eight of the 13 postgraduate residents who were selected in one FGD were in the first and second year of training. The rest of the Obstetricians were in their final year of training. The average length of time in practice was 3 years for the nurses and 7 years for the midwives.

### Positive perspectives on the Partograph

The healthcare workers demonstrated knowledge regarding the utility of the Partograph in obstetrics. Participants drew attention to the importance of the Partograph for handover of care at shift change.

…*“For the wellbeing of the baby and the mother, it can assist you in identifying danger signs during labour. It also lessens the writing work in such critical times, all you need to do is to just plot on the Partograph………” -* **nurses and midwives***.*

*…“With the Partograph, you can be given clear direction on what to do next with the mother. It can help us detect danger signs during labour, monitor the progress of the mother and they lessen the work of writing lots of details, you just plot and fill in small blanks than writing a whole report or case notes…”.-* **nurses and midwives.**

### Negative perspectives and barriers to utilization

Eight themes were identified as negative perspectives to utilization. They included the unavailability of the Partographs, staffing levels and motivation, multitasking required, congestion in the ward, different skill sets and competencies, inadequate monitoring equipment, availability of other methods of monitoring, and status of the referred mother at the time of contact with the health worker.

### Lack of availability of the Partographs

The healthcare workers reported that an inconsistent supply of the Partographs in the hospital. In the few cases where they were available, they were not utilized because they were not seen as part of routine care as expressed in the excerpt:

*“…. not filing or completing the Partograph sometimes feels like a routine, you start on filling in a Partograph then you get an emergency and you abandon that task and you cannot follow up the patient....”* **Obstetrics and Gynecology postgraduate residents.**

### Staffing levels and motivation

Participants noted the dire shortage of human resources creating a time challenge for diligent completion of the Partograph. The low motivation of staff on the labour wards also reduced the feasibility and frequency of use of the Partograph;

*“…the few staff are not motivated sometimes and that is why you will find many Partographs with gaps and are incomplete, no BP, pulse and the like…* **nurses and midwives**

### Multitasking

Participants highlighted the multitasking done by health workers on the ward and how this affected the use of Partographs. The other tasks make Partograph completion an extra task.

*“…Sometimes we are overwhelmed by the number of mothers we attend to. Sometimes the labour ward is too busy and the staff on the ward are really few and thinned so the workload is heavy. That is why you find the ones used are not completed.”* **nurse and midwives**

### Congestion at the hospital wards

Participants noted the maternity and labour wards are highly congested with some mothers even having to labour on the floor as no bed available. The limited number of delivery /theatre rooms available for expectant mothers led to the prolonged need for monitoring.

*“Also there are issues of theatre space so some mothers are kept for long waiting and you find that you end up observing the mother for long and you need more than 1 Partograph to complete” -* ***nurses and midwives.***

*“…There is congestion at the maternity unit – in reality, it is easier to monitor a mother who is on the bed than a mother who is on the floor. It is not easy to monitor a mother on the floor”-* ***FGD with the nurses and midwives.***

### Different health workers with different levels of skill sets and competencies

The healthcare practitioners reported that because Mulago is a teaching hospital, many individuals with different competencies regarding Partograph completion contributed to incompleteness and lack of use. Furthermore, some health workers had little knowledge on the use and completion of Partographs. This is as expressed in the excerpts below.

*“Some of the trainee health workers still have challenges in filling in the entire Partograph so some of them feel that rather than having an incomplete Partograph they are better off not filling it in at all”-* **nurses and midwives.**

### Inadequate equipment and supplies for maternal and fetal monitoring

The lack of needed equipment on the hospital wards for monitoring mothers like stethoscopes, fetoscopes, urine dipsticks, blood pressure machines, was highlighted as a challenge by participants:

*“The other challenge is lack of equipment to cater for the mother, for example, Blood pressure (BP) machine, w), thermometers, few catheters, not enough gloves to do the examinations every required time by the Partograph. The hospital has not enough space and beds to have all the mothers so the environment is not so suitable for example the mothers are on the floor and the like.” -* **nurses and midwives.**

*“Sometimes there are no tools to do the examinations like the fetal scope, BP machine is not there, no urine sticks let alone the Partographs so what is the point of recording some parts”. -* ***nurses and midwives.***

### Availability of other methods of monitoring

The obstetricians and postgraduate doctors training in obstetrics noted that other tools can be used to mitigate the need for the Partograph. However, these other methods were not widely available even at this national referral hospital.

*“Usually we use a CardioTocoGraphy (CTG). This depends on the risk of the mother during labour and we use it for a specific category of the condition of the mother and not all mothers in labour.” -* **Obstetrics and Gynecology postgraduate residents.**

**Status of the mother at the time of contact with health worker** - From the transcripts, the participants revealed that a Partograph may not be completed based upon the situation in which the mother is received by the health worker.

*“Some mothers come to the hospital when they are in the second stage of labour, others come when they are already obstructed so there is no need to plot a Partograph”-* **nurses and midwives.**

## Discussion

The present study revealed a low proportion of 24.6% (88/355) for completed Partographs when all three parameters (fetal, mater and labour monitoring) were considered. Over 60% of the reviewed Partographs were inadequate. This low overall completion rate in our study confirms that completion is not just a problem in rural Uganda as noted in previous rural studies [15, 16].

The finding of low Partograph utilization is similar to a study in 45 public health institutions (5 public hospitals) in Addis-Ababa, Ethiopia which showed a completion rate of 34.4% [23]. A Similar study in Ogun state in Nigeria found comparable low utilization and completion rates [12, 24].

In our study, there was poor documentation of the key maternal (73.9% incomplete), fetal (60.6% incomplete) and labour monitoring (68.3%) incomplete) sections of the Partographs.

This is in line with similar studies in Dar–es- Salaam which showed that only two parameters of the WHO standard Partograph had been completed to slightly over 40% in their review [24, 25]. Other studies in Kenya and Western Uganda also found similar findings of low levels of Partograph completion in the intrapartum stage of pregnancy [15, 26]. The apparent lack of complete documentation of the Partograph parameters in our study may have led to delayed detection and undertaking of urgently required interventions for the mothers and babies leading to high stillbirth rate (results not shown in this paper) in the study period.

The qualitative component of our study provided insights on Partograph utilization. In the Mulago Referral Hospital setting, the health care workers attributed the low completion rate to the heavy workload due to low health care worker numbers, lack of availability of the Partographs in patient charts, lack of equipment, lack of skills and poor attitudes towards its use. Overall healthcare workers were knowledgeable about the usefulness of the Partograph in labour monitoring. This finding is similar to a study done in Nigeria among healthcare workers in the Niger Delta hospital [12]. In our study, the level of training of the study participants (7 years for the midwives, 5 years for the nurses and 1–3 years for the postgraduate trainees in obstetrics and gynaecology) may have contributed to this finding.

The lack of availability of the Partograph was a big hindrance to its utilization in this hospital. This finding is in contrast to another study done among 140 midwives at three hospitals in the Tamale Metropolis area in Ghana where the study participants reported a high level of availability of the Partograph [27]. Similarly, a study in Rujumbura Health sub-district in Southern Western, Uganda found that the various health centers had sufficient numbers of the Partographs although their utilization was low [15]. However, other sites have documented unavailability as a problem for example in Central Ethiopia, Nigeria and South Africa [12, 18, 28]. In our study, the unavailability of Partograph may be due to the high patient numbers served by the hospital, and/or the adequacy of the hospital stationery supply system. Other required essential hospital supplies such as blood pressure cuffs and urine test strips also had gaps.

According to the World Health Organization (WHO), there should be one nurse for every five patients, and one midwife to every four patients on a labour and delivery ward. In Mulago, the nurse or midwife to labouring patient ratio was 1: 15 per shift on the busiest delivery shifts. Uganda has a nurse-patient ratio of 1:11,000 and a doctor-patient ratio of 1:15,000 [29] which is very high but comparable to the nurse to patient ratio of less than 1.15 per 1000 population in other developing countries [30]. This low level of staffing was linked to the low Partograph utilization by the FGD participants in our study. These findings are similar to other studies in African settings on the level of Partograph utilization [3, 14, 31]. In Dar-es-Salaam, Tanzania, similar findings of high workload, made the Partograph completion impractical [25]. This is one of the key bottlenecks to the delivery of quality obstetric care in low to middle income countries. However, this problem is not just in terms of obstetrics care. For example, in 2014/15 Mulago had 1880 staff, this constituted 67% of the staffing positions available leaving a gap of 581 staff posts yet to be filled [32].

In our study, ward congestion also limited Partograph utilization as mothers may be on the floor during labour leading to challenges in the examination and thus Partograph completion. The number of pregnant women coming to Mulago for care and delivery is increasing. During the fiscal year 2014/15, there were 28,759 total antenatal visits, and 39,081 deliveries [32]. In 2015/16 this had risen to 60,902 antenatal visits and 35, 071 deliveries .and a caesarean rate of 26.8% [33] These numbers are overwhelming to the few staff employed by the hospital.

While all the FGD participants saw the value of the Partograph, different health care workers had varying ability to document and utilize the Partograph data. This has been highlighted in other reports as a core need for uptake of Partograph use in resource-limited settings in the world including in previous research in southwestern Uganda where midwives had low knowledge and competencies of the use and how to complete the Partographs [15].

Inadequate equipment and supplies used for generating data for Partograph completion, maternal and fetal monitoring was a major barrier to Partograph utilization in our study. This finding at the national referral hospital is similar to limitations faced in the rural health centers in Rujumbura health sub-district in Uganda indicating a nationwide lack of adequate supplies for health [15].The equipment gaps reported in our e.g. stethoscopes, urine dipsticks, blood pressure machines, among others items needed on the labour wards is not just a problem for maternal care but also reflects unavailability of other key supplies and stock-outs in the hospital in general. Contributors to this situation include poor planning, the delayed repair of broken equipment, poor equipment handling and supply chain breakdown.

All of the above health system challenges that contributed to the low Partograph completion rates undermine the quality of the obstetric care being received by mothers at this high volume health facility. This finding concurs well with similar studies done in the same period [14, 17].

While other modes of monitoring can lessen the need for reliance on the Partograph, for example, *cardiotocography,* their limited availability and cost make this an unrealistic solution to the gap in monitoring problem. Furthermore, different health workers have different competencies in their utilization which could lead to worse labour outcomes if more widely utilized.

There are a number of limitations to our study. The study was conducted in a tertiary referral teaching hospital and this may have introduced patient selection bias. However, as a teaching hospital where highly specialized obstetric care is provided with high-level training, one might have expected optimization of Partograph use compared to a nonteaching hospital. The study was cross-sectional and thus cause-effect relationship was not assessed. Having considered Partograph use throughout the day with no particular predilection to the time of day, our results may have been biased by this. The qualitative study component involved FGD with nurses, midwives and obstetricians but no hospital administrators were included. Their insights on staffing, equipment and supply planning would have enriched the study findings.

## Conclusions

The level of Partograph completion and use in Mulago National Referral Hospital was low. The time required to document, the heavy work load, the health system supply chain challenges, status of mother at presentation, and the Partograph congestion in the hospital, were revealed as challenges to Partograph documentation and utilization. There is a need for further pre-deployment and on-the-job Partograph training to strengthen its utilization in the healthcare settings. However, the crush of the workload and the lack of needed equipment and supplies must also be addressed. The government needs to invest more in healthcare to overcome the challenges to Partograph utilization in order to improve maternal and especially infant outcomes.

## Abbreviations

FGD: Focus Group Discussion
WHO: World Health Organization
